# Temporal trends and demographic patterns in hypertension-related mortality with intracerebral hemorrhage in the United States: 1999–2020

**DOI:** 10.21542/gcsp.2026.3

**Published:** 2026-02-28

**Authors:** Paraag Vashist, Khansa Younus, Sindiri Rohit, Sowkarthick K.S., Abhishek Hanumanpratap Singh Kshatri, Surya Vamsi Gadde

**Affiliations:** 1 Department of General Internal Medicine, Leighton Hospital, Mid Cheshire Hospital, NHS Foundation Trust; 2 Department of Family Medicine, Carmichael Medical Clinic; 3 Department of Internal Medicine, All India Institute of Medical Sciences; 4 Department of Critical Care, St John’s Medical College Hospital, Bangalore; 5 Department of Emergency Medicine, Apollo Hospitals; 6 Department of Internal Medicine, Gayatri Vidya Parishad Institute of Health care and Medical Technology

## Abstract

**Introduction:** Hypertension contributes to cardiovascular mortality, yet its role as an underlying cause with intracerebral hemorrhage (ICH) as a contributing cause is underexplored. Understanding demographic and geographic patterns can guide prevention.

**Aim:** To analyze mortality trends and demographic disparities in deaths with hypertension as the underlying cause and ICH as a contributing cause, using the CDC Multiple Cause of Death (MCD) database from 1999–2020.

**Methods:** Retrospective observational analysis of the CDC MCD database for adults aged ≥25 years in the United States from 1999–2020. Deaths were included if hypertension (I10–I15) was the underlying cause and intracerebral hemorrhage (ICH I61) a contributing cause. Data were stratified by gender, race, geographic region, and place of death. Crude and age-adjusted mortality rates per 1,000,000 and annual percentage change (APC) were calculated. Temporal trends were assessed using Joinpoint software.

**Results**: Among 14,613 deaths (crude rate 3.3 per million), most decedents were male (56.5%) and White (62.8%); Black/African American individuals accounted for 31.2%, Asian/Pacific Islander 5.3%, and American Indian/Alaska Native 0.7%. Most deaths occurred in metropolitan areas (89.4%) and in medical facilities (61.0%), while 25.2% occurred at home. Age-adjusted rates were stable from 1999–2007, declined 2007–2013, then rose 2013–2020. Females showed larger early declines followed by a modest rise, whereas males experienced a later sharper increase. Black/African American decedents had early declines with a subsequent plateau, while White decedents showed smaller early change, followed by decline and later increase.

**Conclusion:** Deaths with hypertension as the underlying cause and ICH as a contributing cause were concentrated among males, White and Black populations, and metropolitan residents, with most deaths in medical facilities. Notably, after a period of decline, mortality rates increased again after 2013, particularly among males and White individuals, underscoring the urgency of renewed prevention efforts.

## Introduction

Hypertension refers to the force exerted by circulating blood against the arterial walls, defined as systolic blood pressure ≥140 mmHg and/or diastolic blood pressure ≥90 mmHg^1^. Treatment across all age groups significantly reduces cardiovascular and cerebrovascular risk^1^. In the U.S. population, the prevalence of hypertension among adults aged over 20 years is 48.4%^2^. The age-adjusted mortality rate increased from 2.8 to 9.4 per 100,000 from 1999 to 2019, reaching 13.9 per 100,000 by 2021, with younger adults experiencing particularly sharp increases in recent years^3^. Globally, hypertension contributes to an estimated 10.4 million deaths annually^4^.

Modifiable risk factors for hypertension include excess body weight, physical inactivity, alcohol use, smoking, diabetes mellitus, obstructive sleep apnea, and chronic kidney disease, while non-modifiable risk factors consist of age, race/ethnicity, and family history^5^.

Intracerebral hemorrhage (ICH) is a type of stroke characterized by bleeding within the brain parenchyma, with or without ventricular extension. Chronic hypertension is the most important risk factor, accounting for over 60% of ICH cases^6^. Hypertensive ICH typically involves deep brain structures, such as the basal ganglia, thalamus, pons, and cerebellum, due to small-vessel pathology, including lipohyalinosis and microaneurysm rupture^7^. Of all strokes in the United States, ICH accounts for approximately 10–20% and represents a particularly devastating subtype, with a 30-day mortality rate approaching 40% and a high rate of long-term disability among survivors^8^.

In the United States, the combined mortality burden of hypertension and ICH remains substantial; however, most prior research has examined these conditions in isolation^9,10^. While ICH is commonly recorded as the underlying cause of death in hemorrhagic stroke cases, death certification practices also recognize clinical scenarios in which long-standing, poorly controlled hypertension is identified as the initiating condition in the causal chain leading to death, with ICH documented as a downstream or contributing event. Such scenarios may include patients with chronic hypertensive vasculopathy who experience fatal intracerebral hemorrhage, where certifying physicians attribute the death primarily to hypertension as the root etiologic driver rather than to the acute hemorrhagic event alone.

Accordingly, in this study, we examine mortality in which hypertension (ICD-10 codes I10–I15) is recorded as the underlying cause of death, and intracerebral hemorrhage (ICD-10 code I61) is listed as a contributing cause of death. This analytic approach provides a hypertension-centric lens that emphasizes deaths attributable to chronic hypertensive disease processes while capturing ICH as a clinically significant and often fatal complication. Although less commonly examined than stroke-centric analyses in which ICH is the underlying cause, this perspective is particularly appropriate for evaluating hypertension-related mortality, as it highlights the role of long-term blood pressure control failure in precipitating fatal cerebrovascular outcomes and allows assessment of temporal and demographic patterns in deaths explicitly attributed to hypertension at the population level.

## Aim and objectives

To evaluate U.S. mortality where hypertensive diseases (I10–I15) were the underlying cause and intracerebral haemorrhage ICH (I61) a contributing cause among adult’s ≥25 years (1999–2020), reporting totals, crude rate per 1,000,000, and distributions by sex, race, 2013 NCHS urban–rural category, and place of death using CDC WONDER MCD.

## Methodology

Data were extracted from the CDC-WONDER Multiple Cause of Death (MCD) database for retrospective analysis^11^. The process of data extraction was performed on 3rd August 2025. The database encompasses death certificate records spanning 1999 to 2020 and was queried to include individuals aged 25 years and older, given the well-documented rarity of hypertension-associated intracranial hemorrhage below this age threshold. As the CDC-WONDER MCD database contains fully de-identified, publicly available data, this study was exempt from formal ethics committee review and institutional approval^12^.

Hypertension (I10–I15) was designated as the underlying cause of death, with intracranial hemorrhage (I61) specified as a contributing multiple cause of death.

Demographic variables included sex (male and female) and race/ethnicity (American Indian or Alaska Native, Asian or Pacific Islander, Black or African American, and White), reflecting the predominant racial groups represented within the United States population. These variables were incorporated to evaluate potential disparities in mortality outcomes across demographic subgroups.

Geographic variables were defined according to the 2013 National Center for Health Statistics (NCHS) urbanization classification scheme, which stratifies counties into six categories: large central metro, large fringe metro, medium metro, and small metro within the metropolitan designation, and micropolitan and non-core areas within the non-metropolitan designation^13^.

Place of death was further classified into five predefined categories: medical facility (inpatient), medical facility (outpatient), decedent’s home, hospice facility, and nursing home^14^. Age-standardized mortality rates were calculated per 1,000,000 population using the United States 2000 standard population as the reference, facilitating valid and accurate temporal comparisons across the study period^15^.

Descriptive statistics were generated for each variable as absolute frequencies and proportions using CDC-WONDER MCD data in accordance with the specified extraction criteria; variables failing to meet these criteria were excluded from the analysis. Temporal trends in age-adjusted mortality rates from 1999 to 2020 were subsequently examined using Joinpoint regression software, which enabled the calculation of annual percentage changes and the identification of statistically significant inflection points over the study period^16^.

## Results

Between 1999 and 2020, the CDC Multiple Causes of Death (MCD) database documented 14,613 fatalities in the United States among individuals aged 25 years and older attributable to hypertension (ICD-10: I10–I15). This analysis also incorporated cases where intracerebral hemorrhage (ICD-10: I61) was recorded as a multiple cause of death, in order to account for cerebrovascular complications associated with hypertension. The crude mortality rate for hypertension was found to be 3.3 per 1,000,000 populations. Deaths not meeting these criteria were excluded from the analysis.

### Demographic and geographical characteristics

Among 14,613 individuals included in the mortality dataset between 1999 and 2020 with hypertension listed as the underlying cause of death and intracerebral hemorrhage as a contributing cause, males represented a greater proportion of deaths than females (8,258 [56.5%] vs. 6,355 [43.5%]), reflecting the consistently higher hypertension and hemorrhagic stroke burden reported among men in epidemiologic studies.

**Table 1:** Baseline demographic characteristics of decedents with hypertension as the underlying cause of death and intracerebral hemorrhage as a contributing cause, United States, 1999–2020. Baseline demographic distribution of decedents aged ≥25 years in whom hypertension (ICD-10 I10–I15) was recorded as the underlying cause of death and intracerebral hemorrhage (ICD-10 I61) as a contributing cause, using CDC WONDER Multiple Cause of Death data (1999–2020). Percentages are calculated within each category.

**Characteristic**	**No. of deaths**	**Percentage (%)**
**Total**	14,613	100
**Sex**		
Male	8,258	56.50
Female	6,355	43.50
**Age, in years**		
25–34	263	1.80
35–44	1,360	9.30
45–54	3,262	22.30
55–64	3,100	21.20
65–74	2,063	14.10
75–84	2,516	17.20
≥85	2,049	14.00
**Race**		
American Indian or Alaska Native	96	0.70
Asian or Pacific Islander	773	5.30
Black or African American	4,561	31.20
White	9,183	62.80
**Residence**		
Metropolitan	13,065	89.40
Non-metropolitan	1,548	10.60

**Table 2:** Age-adjusted mortality rates by demographic characteristics for hypertensionrelated intracerebral hemorrhage mortality, United States, 1999–2020. Age-adjusted mortality rates (AAMR) per 100,000 population were calculated using the direct method and standardized to the 2000 U.S. standard population. Rates represent deaths with hypertension as the underlying cause of death and intracerebral hemorrhage as a contributing cause, derived from CDC WONDER (1999–2020).

**Demographic characteristic**	**AAMR (per 1,000,000)**	**95% Confidence Interval**
**Sex**		
Male	3.8	3.8–3.9
Female	2.4	2.4–2.5
**Race**		
White	2.3	2.3–2.3
Black/African American	8.7	8.5–9
American Indian/Alaska Native	2.3	1.9–2.9
Asian/Pacific Islander	3.7	3.4–4
**Residence**		
Metropolitan	2.1	2.1–2.2
Non-metropolitan	1.3	1.2–1.3

The age distribution demonstrated that mortality clustered predominantly in mid-to-late adulthood, with the highest proportions observed in the 45–54 years (3,262; 22.3%) and 55–64 years (3,100; 21.2%) groups, followed by those aged ≥75 years. Fewer deaths occurred among adults younger than 45 years (total 1,623; 11.1%), indicating that hypertension-related intracerebral hemorrhage remains largely a condition affecting older age strata.

Racial patterns showed that White individuals accounted for the majority of deaths (9,183; 62.8%); however, Black or African American individuals comprised nearly one-third of deaths (4,561; 31.2%), a proportion notably higher than their representation in the U.S. population, suggesting an unequal burden likely driven by longstanding disparities in hypertension control and access to preventive care.

Most deaths occurred in metropolitan settings (13,065; 89.4%), consistent with population clustering in these regions, but potentially also reflective of greater diagnostic capture and access to acute care services where hemorrhagic stroke is more readily identified. Taken together, these demographic patterns illustrate a concentration of mortality among older adults, males, and Black populations, underscoring the need for targeted risk-reduction efforts in high-burden demographic groups (Table 1).

### Mortality rate comparisons

Age-adjusted mortality rates showed substantial demographic variation (Table 2). Overall, mortality was higher among males than females (3.8 vs 2.4 per 100,000), reflecting a greater burden of hypertension-associated ICH deaths in men. Black/African American adults experienced the highest mortality (8.7 per 100,000), nearly four-fold higher than White adults (2.3 per 100,000), indicating persistent racial disparities. Asian/Pacific Islander adults had intermediate mortality (3.7 per 100,000), while American Indian/Alaska Native adults demonstrated lower but imprecise estimates due to small counts (2.3 per 100,000; 95% CI 1.9–2.9).

Mortality was modestly higher in metropolitan than non-metropolitan areas (2.1 vs 1.3 per 100,000), a pattern consistent with the concentration of stroke care and hypertension burden in urban regions. These findings highlight demographic inequalities in hypertension-related ICH mortality and justify subgroup trend analyses.

### Temporal trends

**Figure 1.**
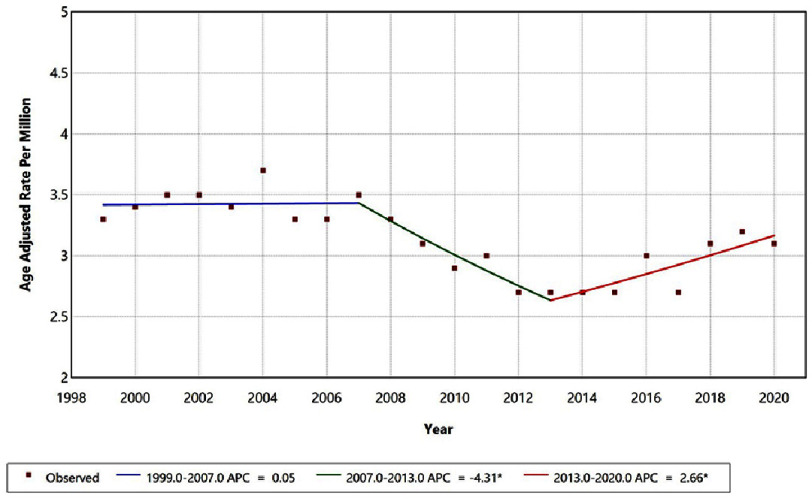
Overall age-adjusted mortality rates among adults aged 25+ in the United States, 1999–2020. *Indicates that the Anuual Percentage Change (APC) is significantly different from zero at alpha=0.05 level.

From 1999–2020, overall age-adjusted mortality due to hypertension with intracerebral hemorrhage as a contributing cause demonstrated three statistically distinct trend segments (Figure 1, Table 3). Mortality rates remained stable between 1999–2007 (APC = 0.05%; 95% CI −1.09 to 3.28; *p* = 0.86), followed by a significant decline from 2007–2013 (APC = −4.31%; 95% CI −9.41 to −2.30; *p* = 0.006). However, this reduction reversed thereafter, with rates increasing significantly from 2013–2020 (APC = 2.66%; 95% CI 0.94 to 7.05; *p* = 0.008), indicating a resurgence in hypertension-related mortality after an earlier period of improvement.

**Table 3:** Joinpoint regression analysis of age-adjusted mortality trends for hypertension-related intracerebral hemorrhage mortality, United States, 1999–2020.

**Cohort**	**Segment**	**Lower** ** Endpoint**	**Upper** **Endpoint**	**APC**	**95% CI**	**P-Value**
Overall	1	1999	2007	0.0512	−1.09–3.28	0.8582
Overall	2	2007	2013	−4.3115^*^	−9.41–2.3	0.0064
Overall	3	2013	2020	2.6573^*^	0.94–7.05	0.0080
Female – 2 Joinpoints	1	1999	2007	−0.5656	−2.35–6.22	0.7267
Female – 2 Joinpoints	2	2007	2012	−6.6956^*^	−12.66–3.08	0.0200
Female – 2 Joinpoints	3	2012	2020	2.3221^*^	0.37–7.25	0.0324
Male – 2 Joinpoints	1	1999	2007	0.4677	−0.65–4.77	0.4183
Male – 2 Joinpoints	2	2007	2015	−2.8157^*^	−8.35–1.52	0.0052
Male – 2 Joinpoints	3	2015	2020	4.9570^*^	1.98–12.5	0.0036
Black or African American – 2 Joinpoints	1	1999	2008	−2.1455	−3.54–2.56	0.1252
Black or African American – 2 Joinpoints	2	2008	2014	−7.2279^*^	−13.58–4.66	0.0164
Black or African American – 2 Joinpoints	3	2014	2020	0.2174	−2.66–9.88	0.7351
White – 2 Joinpoints	1	1999	2008	0.0499	−1.43–1.55	0.9438
White – 2 Joinpoints	2	2008	2013	−3.0030	−7.89–2.14	0.2262
White – 2 Joinpoints	3	2013	2020	3.3401^*^	1.11–5.62	0.0061
Metro – 2 Joinpoints	1	1999	2008	0.1990	−0.73–1.91	0.6107
Metro – 2 Joinpoints	2	2008	2013	−5.4128^*^	−9.4–3.07	<0.0001
Metro – 2 Joinpoints	3	2013	2020	2.0511^*^	0.66–4.54	0.0040
Non-Metro – 2 Joinpoints	1	1999	2014	−2.3813^*^	−3.63–1.12	0.0013
Non-Metro – 2 Joinpoints	2	2014	2018	14.3888	−1.77–33.21	0.0792
Non-Metro – 2 Joinpoints	3	2018	2020	−5.5088	−30.32–28.13	0.6959

**Notes.**

* Indicates that the APC (Annual percentage change) is significantly differ from zero at alpha = 0.05 level.

**Figure 2.**
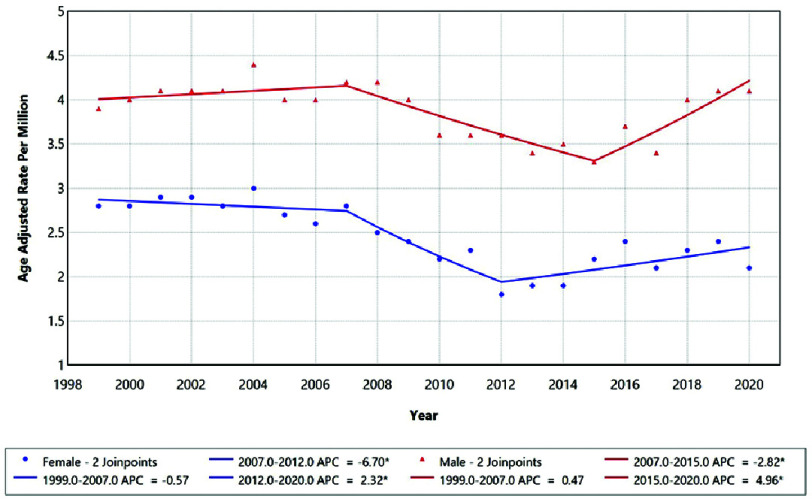
Trends in sex-stratified age-adjusted Mortality rates among adults aged 25+ in the United States, 1999–2020. *Indicates that the Anuual Percentage Change (APC) is significantly different from zero at alpha=0.05 level.

Sex-stratified analyses revealed divergent patterns between males and females (Figure 2). In females, mortality trends were initially stable from 1999–2007 (APC = −0.57%; 95% CI −2.35 to 6.22; *p* = 0.73), followed by a significant decline from 2007–2012 (APC = −6.70%; 95% CI –12.66 to −3.08; *p* = 0.02), and subsequently a significant rise during 2012–2020 (APC = 2.32%; 95% CI 0.37 to 7.25; *p* = 0.03). In contrast, males experienced stable rates from 1999–2007 (APC = 0.47%; 95% CI −0.65 to 4.77; *p* = 0.42), a significant decline between 2007–2015 (APC = −2.82%; 95% CI −8.35 to −1.52; *p* = 0.005), and a subsequent sharper increase from 2015–2020 (APC = 4.96%; 95% CI 1.98 to 12.50; *p* = 0.004). These contrasting trajectories were statistically non-parallel (*p* = 0.010), confirming sex-specific differences in temporal mortality patterns (Table 4).

**Table 4:** Joinpoint comparability analysis of temporal trends.

Model	Maximum Joinpoints (Kmax)	Test	Numerator DF	Denominator DF	Permutations	*P*-value	Interpretation
Sex-Combined Joinpoint model	2	Test of Parallelism	5	32	4,500	0.0100	Trends differ significantly between males and females
Urbanization-Combined Joinpoint model	2	Test of Parallelism	5	32	4,500	0.0036	Trends differ significantly between Metro and Non-Metro
Race-Combined Joinpoint model	2	Test of Parallelism	5	32	4,500	0.0002	Trends differ significantly between Black or African American and White

**Notes.**

* Comparison for American Indian or Alaska Native and Asian or Pacific Islander cannot be done as data was suppressed.

**Figure 3.**
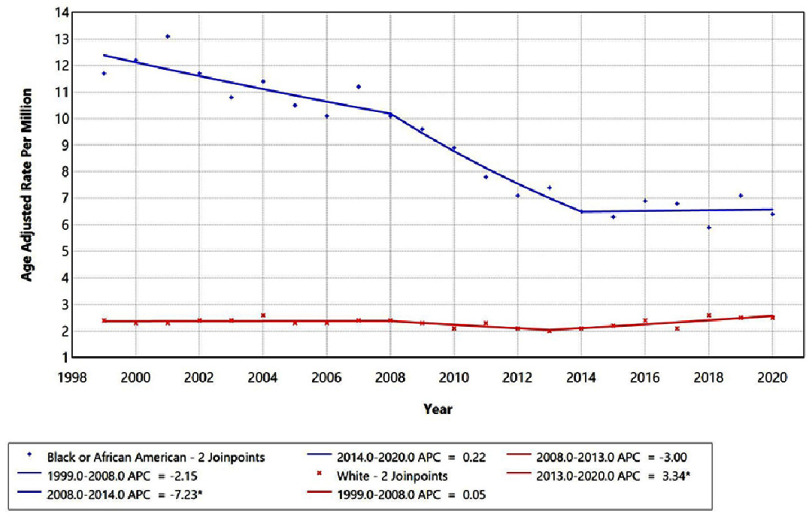
Trends in age-adjusted mortality rates stratified by race among adults aged 25+ years in the United States, 1999 to 2020. *Indicates that the Anuual Percentage Change (APC) is significantly different from zero at alpha=0.05 level. Temporal trends for American Indian/Alaska Native and Asian or Pacific Islander are not displayed due to data suppression for counts <10, limiting reliable trend analysis.

Race-stratified analyses further demonstrated heterogeneity in mortality trends (Figure 3). Among Black/African American adults, mortality declined during 1999–2008 (APC = −2.15%; 95% CI −3.54 to 2.56; *p* = 0.13) and more sharply between 2008–2014 (APC = −7.23%; 95% CI –13.58 to −4.66; *p* = 0.016), before stabilizing during 2014–2020 (APC = 0.22%; 95% CI −2.66 to 9.88; *p* = 0.74). Among White adults, mortality was stable from 1999–2008 (APC = 0.05%; 95% CI −1.43 to 1.55; *p* = 0.94), followed by a non-significant decline during 2008–2013 (APC = −3.00%; 95% CI −7.89 to 2.14; *p* = 0.23), and a significant rise between 2013–2020 (APC = 3.34%; 95% CI 1.11 to 5.62; *p* = 0.006). Comparable trend testing confirmed significant non-parallelism between Black and White groups (*p* = 0.0002; Table 4), indicating persistent racial disparities and differing temporal dynamics. Trends for American Indian/Alaska Native and Asian/Pacific Islander adults could not be assessed due to data suppression for small counts (<10).

Urban–rural patterns also differed significantly over time (*p* = 0.0036; Table 4), with metropolitan areas showing a significant decline from 2008–2013 (APC = −5.41%; 95% CI −9.40 to −3.07; *p* < 0.001) followed by an increase from 2013–2020 (APC = 2.05%; 95% CI 0.66 to 4.54; *p* = 0.004). In contrast, non-metropolitan trends were more variable, with an initial decline followed by a short-term rise and subsequent non-significant decrease; however, suppression of unstable estimates limited interpretation. Taken together, these results highlight a nationwide rebound in hypertension-related intracerebral hemorrhage mortality after 2013, with significant differences by sex, race, and urbanicity.

**Figure 4.**
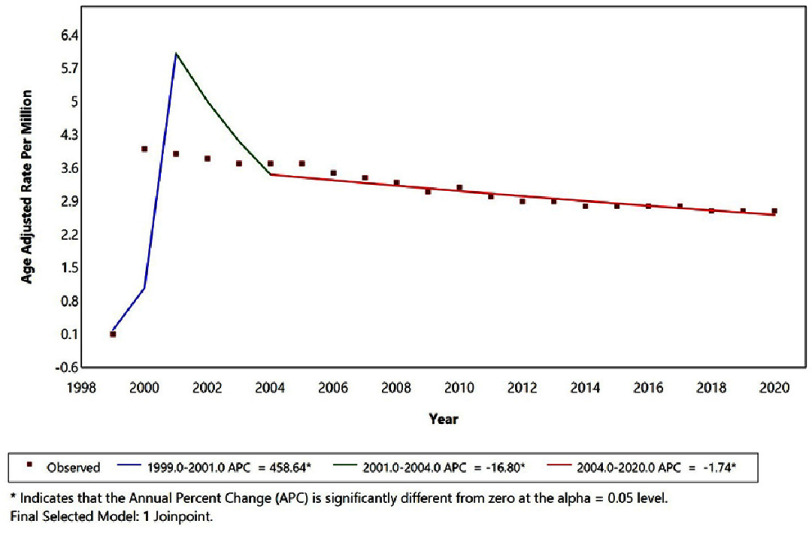
Temporal trends in age-adjusted mortality with intracerebral hemorrhage as the underlying cause of death and hypertension as a contributing cause, United States, 1999–2020.

In a complementary analysis examining deaths in which intracerebral hemorrhage was recorded as the underlying cause of death and hypertension as a contributing cause, age-adjusted mortality trends differed from those observed in the primary analysis (Figure 4). Mortality rates increased sharply between 1999 and 2001 (APC = +458.64%; 95% CI not shown; *p* < 0.05), followed by a significant decline from 2001 to 2004 (APC = −16.80%; *p* < 0.05). From 2004 through 2020, a continued but more gradual decline in age-adjusted mortality was observed (APC = −1.74%; *p* < 0.05). No post-2013 increase in mortality rates was identified in this stroke-centric analysis, indicating a sustained downward trend when intracerebral hemorrhage was specified as the underlying cause of death and hypertension was listed as a contributing condition.

## Discussion

This population-based study demonstrates important long-term trends in mortality attributed to hypertension (ICD-10 codes I10–I15) with intracerebral hemorrhage (ICH; ICD-10 code I61) as a contributing cause of death among United States adults aged 25 years and older over the two-decade period from 1999 to 2020.

Over these 22 years, 14,613 deaths were identified, with a distinct three-phase temporal pattern: an initial period of stability, a significant decline between 2007 and 2013, followed by a statistically significant increase in mortality after 2013. This post-2013 reversal represents the most clinically and public health–relevant finding of the study.

### Mortality reversal after 2013

The resurgence in age-adjusted mortality observed after 2013 likely reflects a convergence of biological, clinical, and healthcare-system factors. Earlier declines in mortality may be attributable to improved hypertension awareness, treatment, and acute stroke care, consistent with prior national trends^17,18^. These favorable trends also coincided with broader healthcare system reforms that expanded insurance coverage and access to preventive and chronic disease care, which have been associated with improvements in population-level health outcomes and reductions in mortality over time^19^.

However, sustained long-term blood pressure control remains challenging, particularly in populations with high cardiometabolic risk burdens and adverse social determinants of health. Even with improved access to care, gaps in hypertension treatment adherence, continuity of care, and effective blood pressure control persist, especially among socioeconomically disadvantaged groups^20^. Chronic poorly controlled hypertension leads to maladaptive shifts in cerebral autoregulation, structural weakening of small penetrating arteries, and development of Charcot–Bouchard microaneurysms, all of which increase susceptibility to intracerebral hemorrhage^21–23^.

The observed post-2013 reversal may also coincide with broader changes in hypertension management paradigms, evolving clinical thresholds for treatment intensity, and the increasing prevalence of obesity, diabetes, and chronic kidney disease—factors known to amplify long-term cerebrovascular risk^24,25^. In parallel, the expanding use of anticoagulant therapies in aging hypertensive populations may increase the severity and fatality of hemorrhagic events when blood pressure control is suboptimal^24,26,27^. Although these therapies reduce thromboembolic risk, their interaction with poorly controlled hypertension may disproportionately worsen outcomes once intracerebral hemorrhage occurs.

Importantly, the complementary analysis in which ICH was treated as the underlying cause demonstrated a continued long-term decline in stroke-centric mortality, suggesting that the observed post-2013 increase is specific to deaths attributed primarily to hypertension rather than hemorrhagic stroke overall. This divergence underscores the importance of cause-of-death attribution and supports prior evidence showing that insurance expansion and improved access to cardiovascular care are associated with reductions in overall cardiac and cerebrovascular mortality outside acute care settings, even when condition-specific or attribution-based mortality trends differ^28^.

### Racial disparities

Marked racial disparities were evident throughout the study period. While White individuals accounted for the highest absolute number of deaths, Black/African American adults experienced substantially higher age-adjusted mortality rates. This finding is consistent with prior literature demonstrating an earlier onset of hypertension, greater disease severity, and poorer blood pressure control among Black populations^29,30^. Importantly, these disparities cannot be attributed solely to biological susceptibility and instead reflect the cumulative effects of adverse social determinants of health, including socioeconomic disadvantage, chronic psychosocial stress, and neighborhood-level inequities.

Structural racism plays a critical role in shaping long-term cardiovascular risk through mechanisms such as residential segregation, reduced access to healthy food environments, limited opportunities for physical activity, and sustained exposure to stressors that contribute to poor hypertension control. Differences in healthcare access, continuity of care, and quality of hypertension management—including delayed diagnosis, undertreatment, and reduced access to specialty stroke care—may further increase vulnerability to catastrophic cerebrovascular outcomes among Black adults. Prior population-based studies have demonstrated that Black individuals experience a disproportionately higher risk of intracerebral hemorrhage and stroke-related mortality, even after accounting for traditional risk factors^31,32^.

Our findings align with a broader body of evidence documenting persistent racial inequities in hypertension prevalence, treatment, and cardiovascular mortality in the United States^26–28^. Interpretation of trends among American Indian/Alaska Native and Asian/Pacific Islander populations was limited by data suppression due to small counts, underscoring ongoing gaps in national surveillance systems for smaller racial and ethnic groups and highlighting the need for improved data capture to better understand disparities in these populations.

### Urban–rural differences

Geographic disparities were also evident, with the majority of deaths occurring in metropolitan areas. While urban populations may have a higher burden of cardiometabolic risk factors and social stressors, differences in mortality patterns between metropolitan and non-metropolitan areas likely extend beyond lifestyle factors alone. Access to timely emergency medical services, hospital availability, and the distribution of comprehensive stroke centers vary substantially by geography and may critically influence outcomes following intracerebral hemorrhage^33,34^.

Non-metropolitan populations often face longer emergency medical services response and transport times, fewer hospitals with advanced stroke capabilities, and limited access to specialized neurocritical care, all of which may adversely affect survival following acute cerebrovascular events^35^. Conversely, higher mortality observed in metropolitan areas may reflect greater disease burden, population density, and differential healthcare utilization patterns, including higher rates of diagnostic recognition and cause-of-death attribution.

Additionally, regional variation in death certificate completion practices may influence whether hypertension and intracerebral hemorrhage are recorded as underlying versus contributing causes of death, potentially contributing to observed geographic differences^36^. Together, these findings suggest that both urban and rural populations require tailored, context-specific strategies addressing healthcare infrastructure, equitable access to emergency stroke care, and long-term hypertension prevention and management.

## Limitations

This study has limitations inherent to analyses of death certificate–based data. Cause-of-death reporting may be subject to misclassification bias due to variability in physician certification practices and inaccuracies in documenting the causal sequence of death. Although ICD-10 codes were applied consistently, changes in diagnostic awareness, documentation, and coding practices over time may have influenced observed trends. As a population-level analysis, findings are susceptible to ecological fallacy and cannot be interpreted as individual-level causal associations. The absence of individual clinical information—including blood pressure control, medication adherence, comorbidities, anticoagulant use, and access to acute care—limits adjustment for confounders. Small cell counts and data suppression (<10 deaths) restricted reliable estimation for certain racial groups and limited calculation of subgroup-specific trend and comparative statistics. Declining autopsy rates may have reduced diagnostic certainty, and COVID-19–related disruptions in 2020 may have affected mortality reporting and completeness.

## Conclusions

From 1999 to 2020, 14,613 deaths occurred in the United States in which hypertensive disease was recorded as the underlying cause of death and intracerebral hemorrhage as a contributing cause, corresponding to a crude mortality rate of 3.3 per 1,000,000 adults. Mortality was disproportionately concentrated among men, metropolitan residents, and White and Black populations, with most deaths occurring in inpatient medical facilities. After a period of decline between 2007 and 2013, age-adjusted mortality rates increased significantly thereafter, indicating a reversal of earlier gains in hypertension-related mortality reduction.

This resurgence in mortality is clinically and public-health relevant, as it suggests gaps in long-term blood pressure control, risk-factor modification, and continuity of care despite advances in antihypertensive therapy and stroke management. Clinically, these findings underscore the need for earlier identification of high-risk individuals, sustained hypertension control across the life course, and aggressive management of comorbid conditions that increase susceptibility to intracerebral hemorrhage. From a public health perspective, targeted prevention strategies—including population-level blood pressure screening, improved adherence to hypertension treatment guidelines, and focused interventions in high-burden demographic and metropolitan populations—are essential to prevent further increases in mortality.

Future efforts should prioritize strengthening primary prevention, improving equitable access to chronic cardiovascular care, and monitoring hypertension-related outcomes using cause-of-death frameworks that capture both underlying disease and fatal cerebrovascular complications. Addressing the observed resurgence is critical to reversing current trends and reducing preventable deaths attributable to hypertension and intracerebral hemorrhage.
